# Pediatric hydrocephalus management at a major neurosurgical referral center in Kumasi, Ghana

**DOI:** 10.1007/s00381-025-06960-9

**Published:** 2025-09-29

**Authors:** Joseline Haizel-Cobbina, Christian Coompson, Samuel Addy, Kwadwo Darko, Derrick Obiri-Yeboah, Kwaku Ampofo, Dickson Bandoh, Benedict Owusu, Anthony Lamina, Jeffrey P. Blount, James M. Johnston, Michael C. Dewan, Frank Nketiah-Boakye

**Affiliations:** 1 Department of Neurological Surgery, Vanderbilt University Medical Center, Nashville, TN USA; 2 Vanderbilt Institute for Global Health, Vanderbilt University Medical Center, 2525 West End Avenue, Nashville, TN 37203 USA; 3 Department of Surgery, Neurosurgery Unit, Komfo Anokye Teaching Hospital, Kumasi, Ghana; 4 CURE International, Grand Rapids, MI USA; 5 Department of Surgery, Neurosurgery Unit, Korle Bu Teaching Hospital, Accra, Ghana; 6 Department of Neurosurgery, Mayo Clinic College of Medicine and Science, Rochester, MN USA; 7 Division of Pediatric Neurosurgery, Childrens of Alabama, University of Alabama at Birmingham, Birmingham, AL USA

**Keywords:** Hydrocephalus, CSF diversion, Treatment failure, Travel distance, Surgery wait time, Late presentation

## Abstract

**Introduction:**

This study aims to determine the presentation and treatment of pediatric hydrocephalus at a major neurosurgical referral center in Ghana and compare outcomes between ventriculoperitoneal shunt (VPS) and endoscopic third ventriculostomy (ETV) with or without choroid plexus cauterization (CPC).

**Methods:**

An ambispective study of pediatric hydrocephalus patients managed at Komfo Anokye Teaching Hospital (KATH), Kumasi-Ghana, was performed between 2019 and 2023. Patient demographics, etiology, surgical details, and outcomes were collected and analyzed. The primary outcomes were treatment failure and time to failure (TTF).

**Results:**

A total of 325 patients were included in the study. Median age at presentation was 6.9 months (IQR 1.4, 36.4), and 52% were male. The predominant etiologies were myelomeningocele-associated hydrocephalus (23%) and post-infectious hydrocephalus (21%). Macrocephaly (89%) and bulging fontanelle (73%) were the frequent clinical presentations. Of the 247 patients (76%) who underwent CSF diversion, 70% underwent VPS, 19% underwent ETV, and 11% underwent ETV/CPC. The median time from presentation to surgical management was 29 (IQR 8, 83) days. Thirty-day surgical mortality was 8%. Median postoperative length of stay was 7 days (IQR 5, 14). Six percent of VPS patients and 9% of ETV ± CPC patients required re-intervention with a median TTF of 2.5 months and 1.5 months respectively (*p* = 0.40). At a median follow-up duration of 15 months (IQR 6, 29), 69% of patients were alive.

**Conclusion:**

There is a substantial pediatric hydrocephalus burden at KATH in Kumasi-Ghana. ETV ± CPC and VPS demonstrate comparable treatment success. Public health interventions aimed at prevention and provision of timely neurosurgical care may help reduce disease burden and improve outcomes.

## Introduction

Hydrocephalus is in the most commonly encountered surgical condition affecting pediatric patients [1]. It affects approximately 1–2 in every 1000 live births, with a higher burden of cases reported in low- and middle-income countries (LMICs) [2]. An estimated 100,000–200,000 pediatric hydrocephalus cases occur annually in Africa [1]. A Ghanaian hospital-based study estimated the prevalence of pediatric hydrocephalus to be 110 per 1000 pediatric population [3].

Without appropriate treatment, hydrocephalus can lead to significant neurological deficits and increased mortality [4, 5]. The high disease burden in LMICs, including African countries, coupled with the scarcity of neurosurgeons and neurosurgical infrastructure, poses a significant challenge to hydrocephalus care, leading to poor outcomes in LMICs [6, 7].

Despite advances in hydrocephalus treatment options including endoscopic approaches and protocoled guidelines, data on hydrocephalus treatment in the Ghanaian context is lacking. This study aims to assess the treatment outcomes for hydrocephalus and as well as the challenges encountered in accessing hydrocephalus care at the Komfo Anokye Teaching Hospital over a 5-year period. A clearer understanding of the current landscape may inform healthcare policy, enhance clinical practices, and ultimately improve outcomes for affected children.

## Methods

### Design and setting

We conducted an ambispective cohort study of pediatric hydrocephalus patients managed at Komfo Anokye Teaching Hospital (KATH), Kumasi-Ghana, between 2019 and 2023. KATH is the second largest public referral hospital in Ghana with a 1200-bed capacity and 3-bed pediatric intensive care unit (PICU) [8]. Pediatric hydrocephalus care is currently available in five hospitals across three regions in Ghana [9]. Three (Cape Coast Teaching Hospital, Greater Accra Regional Hospital, and 37 Military Hospital) out of the five hospitals are only able to offer VPS insertion, and two hospitals (KATH and Korle Bu Teaching Hospital) provide both VPS and endoscopic treatment.

The KATH Hydrocephalus Spina Bifida (HSB) database was established as part of the CURE Neuro Care Program implemented through a partnership between CURE International and KATH [10]. The CURE Neuro Care Program Coordinator provides operational support for the program by maintaining the Hydrocephalus and Spina Bifida (HSB) database, providing counseling to patients and caregivers, and conducting follow-up home visits and telephone calls. It is currently one of the five hospitals in Ghana which provides pediatric hydrocephalus care.

### Data collection

Data collected included demographic information, etiology of hydrocephalus, clinical presentation, index CSF diversion procedure, need for re-intervention, treatment outcomes, and follow-up.

The primary outcome measures were hydrocephalus treatment failure and time to failure (TTF). Treatment failure was defined as the need for a repeat CSF diversion procedure at any point following the index CSF diversion or death attributed to hydrocephalus. Time to failure was measured as the duration between the index CSF diversion procedure and the repeat CSF diversion procedure. Secondary outcomes of interest included postoperative complications and surgical mortality. Surgical mortality was defined as death within 30 days of the CSF diversion procedure. Comparative analyses of outcomes were conducted between the two treatment groups: VPS and ETV ± CPC.

### Statistical and geospatial analysis

Descriptive statistics were used to summarize data. Categorical variables were reported as frequencies and percentages. Continuous variables were reported as median and interquartile ranges (IQR) for skewed data distributions and means with standard deviation for normally distributed data. In treatment group analysis, categorical data were compared using Fisher’s exact test, and continuous data were compared using independent *t*-tests.

Univariate linear regression analyses were performed to assess the relationship between independent patient travel distances to KATH and (1) disease severity at presentation using head circumference percentile as a proxy measure and (2) timing of surgical intervention. Univariate logistic regression analyses were conducted to evaluate the association between travel distance and (1) the likelihood of patients receiving surgical intervention and (2) treatment outcome. Missing data in the dataset were addressed using the k-nearest neighbors imputation method (*k* = 5) prior to regression analysis. Results were reported as a beta coefficient or ORs (odds ratios) with corresponding 95% confidence intervals (CI) and *p*-values. Statistical significance was set at a two-tailed *p* < 0.05.

A geospatial analysis and visualization of the regional origins of patients were conducted to assess the travel distances between KATH and the locations of patients’ residence. The hospital’s geographic coordinates were manually defined, while patient location data, including longitude and latitude, were auto populated. The Haversine formula [11], implemented through the *geosphere* package in R studio, was used to compute geodesic distances in kilometers (km) between the hospital and each patient’s location. The results of this analysis were represented on a Ghana map using the *ggplot* package in R studio.

### Ethical approval

Institutional Review Board (IRB) approval was obtained from KATH IRB and Vanderbilt University Medical Center (VUMC) IRB.

## Results

### Demographics and clinical presentation

The study cohort comprised of 325 patients including 169 (52%) males and 156 (48%) females. The median age at presentation was 6.9 months (IQR 1.4, 36.4). The predominant etiologies were myelomeningocele-associated hydrocephalus (MMC-associated) (23%), post-infectious hydrocephalus (PIH) (21%), and tumor-related hydrocephalus (15%) (Table 1).

**Table 1:** Demographics, clinical presentation, and initial treatment

Variables	
***Demographics***	
** Age at presentation (in months)**	6.9 (1.4, 36.4)
***Gender***	
Female	156 (48%)
Male	169 (52%)
***Etiology***	***N* = *325***
Post-infectious	67 (20.6%)
Myelomeningocele associated	76 (23.4%)
Encephalocele-related	5 (1.5%)
Tumor-related	49 (15.1%)
Dandy-Walker complex	35 (10.8%)
Aqueductal stenosis	38 (11.7%)
Congenital idiopathic	20 (6.2%)
Hydranencephaly	12 (3.7%)
Holoprosencephaly	6 (1.8%)
Cyst-related*, e.g., congenital cyst, arachnoid cyst	3 (0.9%)
Septated/loculated hydrocephalus^⸷^	2 (0.6%)
Other (unspecified)	12 (3.7%)
***Clinical presentation***	***N* = *325***
Macrocephaly	290 (89%)
Vomiting	107 (31.0%)
Poor feeding	86 (24.9%)
Seizures	52 (15.1%)
Ocular abnormalities	66 (19.1%)
Irritability	49 (14.2%)
Lethargy	32 (9.3%)
Failure to thrive	24 (7.0%)
Headache	21 (6.5%)
Fontanelle	
*1) Above the bone*	237 (72.9%)
*2) At or below the bone*	33 (10.2%)
*3) Sutures closed*	48 (14.7%)
Head circumference measurement (cm)	50.9 ± 9.2
Head circumference percentile	94.9 ± 11.3
***Initial CSF diversion procedure***	***N* = *247***
VPS insertion	172 (69.6%)
ETV	47 (19.0%)
ETV/CPC	28 (11.3%)

Values are presented as the number of patients (%) or median (Quartile 1, Quartile 3) unless stated otherwise

^*^Includes patients who had intracranial congenital cysts such as arachnoid cysts

⸷Septated/loculated hydrocephalus (not related to infection or hemorrhage)

In terms of clinical presentation, macrocephaly (89%) was the most prevalent feature followed by bulging fontanelle (73%), vomiting (31%), poor feeding (25%), and ocular abnormalities (19%). The mean ± SD preoperative head circumference measurement and percentile for the overall cohort were 50.9 ± 9.2 cm and 94.9 ± 11.3 respectively. Table 1 details the demographic and clinical presentation for the entire cohort.

### Hydrocephalus treatment

Of the 325 patients, 247 (76%) underwent CSF diversion procedures with VPS insertion being the most common intervention (69.6%). ETV and ETV/CPC were performed in 19.0% and 11.3% of cases, respectively. The median age for the overall cohort at the time of CSF diversion was 12 months (IQR 4, 49). The median age at the time of CSF diversion was statistically similar for both VPS and ETV ± CPC groups (13.0 mo vs 12.6 mo, *p* = 0.13). There were no statistically significant differences in mean head circumference (53.7 cm vs. 52.7 cm, *p* = 0.381) or percentile (96.3 vs. 95.4, *p* = 0.554) between the two treatment groups.

The median time from presentation to CSF diversion was 29 (IQR 8, 83) days for the entire cohort. While the median time from presentation to CSF diversion was slightly longer in the ETV/CPC group (34.5 days) compared to the VPS group (23 days), this difference was not statistically significant (*p* = 0.486) (Table 2).

**Table 2:** Comparison of etiology, hydrocephalus metrics, and in-hospital outcomes between VPS and ETV±CPC groups

Variable	Procedure		*p*-value
	VPS (*n*=172)	ETV±CPC (*n*=75)	
**Age at intervention (in months)**	13 [4.0, 71.8]	12.6 [5.5, 41.1]	0.130
**Etiology**			
Post infectious	44 (25.6%)	13 (17.3%)	0.190
Tumor-related	36 (20.9%)	10 (13.3%)	0.213
Spina-bifida associated	16 (9.3%)	6 (8.0%)	0.813
Dandy-Walker complex	18 (10.5%)	18 (24%)	0.010
Aqueductal stenosis	14 (8.1%)	16 (21.3%)	0.006
Congenital idiopathic	16 (9.3%)	3 (4%)	0.198
Encephalocele-related	5 (2.9%)	3 (4%)	0.702
Hydranencephaly	8 (4.7%)	4 (5.3%)	0.759
Holoprosencephaly	2 (1.2%)	1 (1.3%)	1.000
Cyst	2 (1.2%)	1 (1.3%)	1.000
Septated/loculated hydrocephalus	1 (0.6%)	0 (0%)	1.000
Unspecified	10 (5.8%)	0 (0%)	0.035
**Hydrocephalus metrics**			
Preoperative head circumference (cm)*	53.7 ± 8.3	52.7 ± 8.1	0.381
Preoperative head circumference Percentile*	96.3 ± 9.4	95.4 ± 10.6	0.554
**Time from presentation to CSF diversion (in days)**	23 [6.3, 80.8]	34.5 [15, 91.5]	0.486
**Post-operative LOS (in days)**	7 [4.5, 14]	8 [5.0, 14.0]	0.999
**Surgical mortality**	12 (7.0%)	8 (10.7%)	0.322

Values are presented as the number of patients (%) or median [IQR] unless stated otherwise*Values reported as Mean ± SD

In this cohort, 78 (24%) out of the 325 patients did not receive surgical intervention, out of which seven died while awaiting the CSF diversion procedure.

### Postoperative hospital stay and complications

Twenty-four patients (10%) experienced seizures in the postoperative period, four patients (2%) had a surgical site infection, four patients (2%) had pressure ulcers, and three (1%) patients had a CSF leak. In the VPS group specifically, ten patients (6%) developed shunt infections, four patients (2%) had exposed shunts, and two patients (1%) were diagnosed with bowel perforation in the early postoperative period. The overall surgical mortality rate following CSF diversion was 8% with similar surgical mortality rates between VPS and ETV ± CPC groups (7% vs 11%, *p* = 0.33) (Table 2). From the medical records, of the 20 patients who died within 30 days of surgery, 8 (40%) were hydrocephalus-related and 12 (60%) were related to medical comorbidities. The eight hydrocephalus-related deaths were due to shunt malfunction (five), shunt infection (one), ventriculitis (one), and intraventricular hemorrhage (one). Deaths related to medical comorbidities included sepsis (4), pneumonia (2), epilepsy and status epilepticus (3), cardiopulmonary arrest (2), and severe malaria (one). The median postoperative length of stay was 7 days (IQR 5, 14). Ten patients required hydrocephalus-related hospital readmissions following discharge.

### Treatment failure and time to failure

Re-intervention rates were comparable between the two groups, with 11 (6%) VPS patients and 7 (9%) ETV ± CPC patients requiring a repeat CSF diversion procedure. The median TTF did not differ significantly between VPS and ETV ± CPC groups (2.5 months vs. 1.5 months, *p* = 0.40) (Table 3).

**Table 3:** Hydrocephalus re-intervention after failure of index procedure

Variable	Procedure		*p*-value
	VPS (*n*=172)	ETV±CPC (*n*=75)	
**Re-intervention for hydrocephalus**	11 (6%)	7 (9%)	1.000
**Type of Re-intervention**			0.431
Shunt	10 (91%)	7 (100%)	
ETV	1 (9%)	0 (0%)	
**TTF (months)**	2.4 [2, 7]	1.5 [1.1, 2.0]	0.403
**Alive at last follow-up**	121 (70%)	51 (68%)	0.764
**Length of follow-up (months)**	17 [4, 29]	12 [6, 28]	0.285

*Values are presented as the number of patients (%) or median [IQR]*

### Follow-up

Follow-up information was available for 316 (92%) patients, defined as those who had at least one follow-up visit either in the neurosurgery clinic or via another avenue. At a median follow-up duration of 15 months (IQR 6, 29), 69% were alive at last follow-up. Stratifying by treatment groups, 70% of patients in the VPS group and 68% of patients in the ETV ± CPC were alive at last follow-up.

### Travel distance

**Fig. 1:**
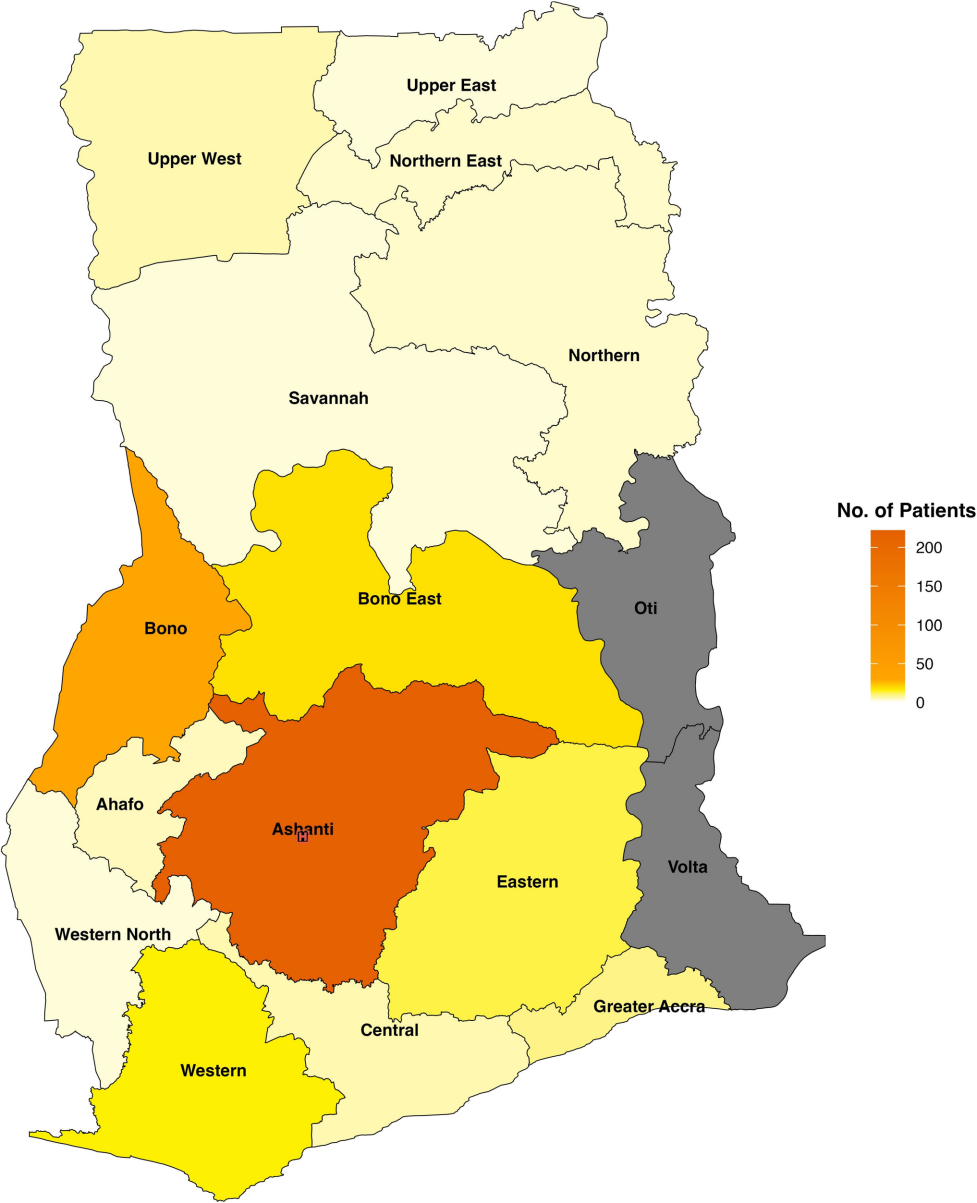
Geographic distribution of patients. Map illustrates the number of patients from various regions across Ghana, with the highest concentration from the Ashanti Region. *Two patients referred from outside Ghana (Sierra Leone and Côte d’Ivoire) not shown on map

**Fig. 2:**
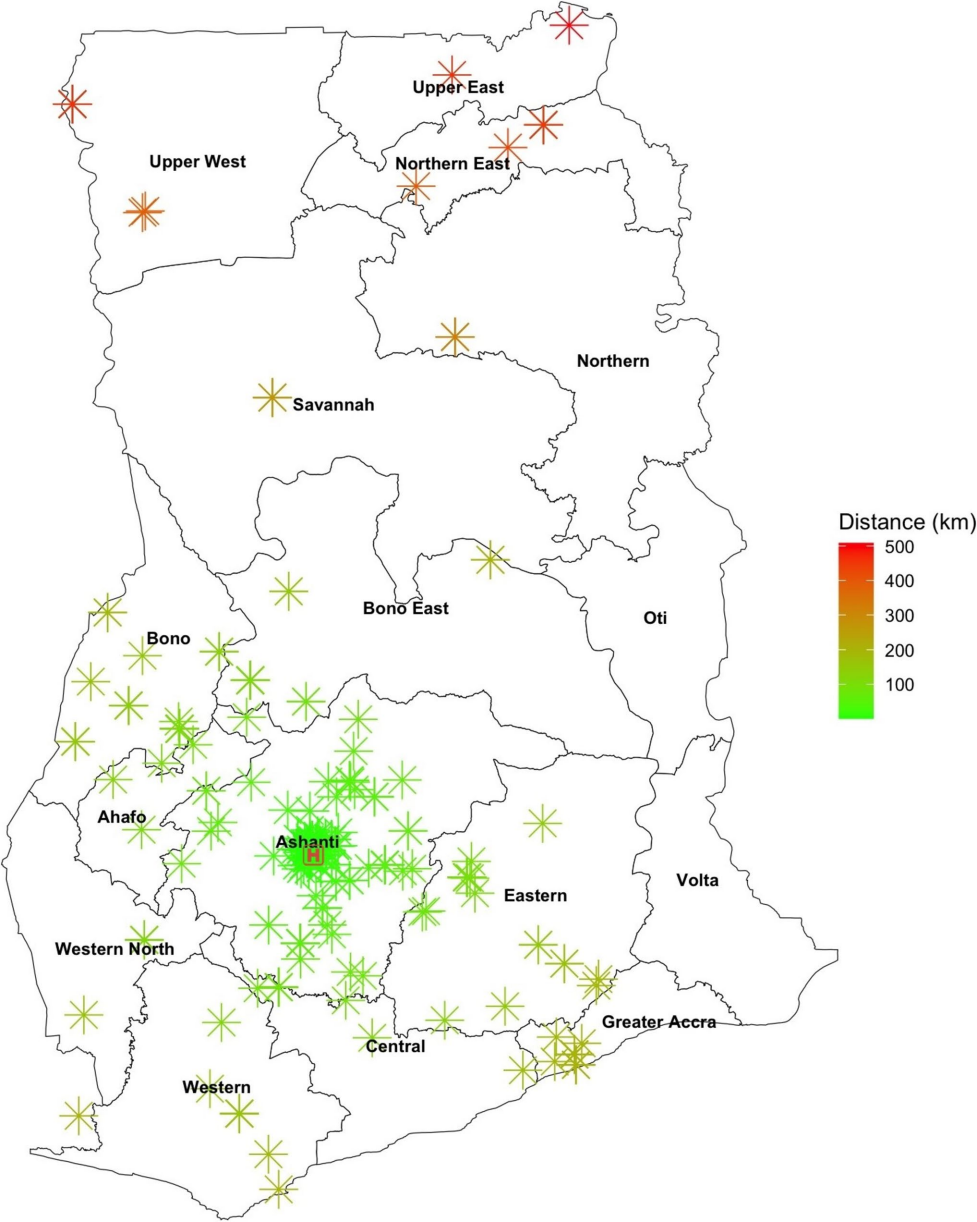
Geographic representation of patient locations and proximity to KATH. *Location of two patients referred from Sierra Leone and Côte d’Ivoire not shown on map

Among the 16 regions in Ghana, KATH provided pediatric hydrocephalus care to patients from 14 regions, with the majority (65%) from the Ashanti region (Fig. 1). The overall median distance traveled to seek pediatric hydrocephalus treatment at KATH was 46.5 km (IQR 4.4, 105) (Fig. 2). Thirty-six percent (118/325) of patients lived greater than 100 km from KATH. The longest distance traveled within Ghana was 508.3 km from Bawku in the Upper East Region of Ghana. One patient each was referred to KATH from Sierra Leone and Côte d'Ivoire.

Univariate linear analyses evaluating associations between travel distance to KATH and clinical parameters showed no significant association between disease severity at initial presentation (*β* = 0.005, 95% CI −0.005 to 0.015, *p* = 0.349) or time to surgery (*β* = −0.04, 95% CI −0.15 to 0.06, *p* = 0.448). Similarly, in the logistic regression, no significant associations were found between travel distance to KATH and surgical intervention (OR = 0.998, 95% CI 0.996 to 1.001, *p* = 0.143) or treatment outcome (OR = 1.001, 95% CI 0.999 to 1.004, *p* = 0.298).

## Discussion

The current study is the first to comprehensively describe the burden and treatment outcomes of pediatric hydrocephalus at KATH, Kumasi-Ghana. The data highlights a substantial disease burden over the 5-year period. Fifty-nine percent of the cases were attributed to myelomeningocele-associated hydrocephalus, post-infectious hydrocephalus (PIH), and tumor-related hydrocephalus—findings consistent with other African studies [5, 12–14]. About 76% of patients underwent CSF diversion at a median time of 29 days, and 69% of patients were alive at the time of last follow-up. These findings underscore ongoing challenges in providing timely and high-quality pediatric neurosurgical care in Ghana.

Macrocephaly, bulging fontanelle, and vomiting were the most common presentations among patients, which suggests that the majority of patients present with severe disease. Although the study did not capture symptom duration prior to presentation at KATH, a previous research report indicates an average delay of 7 months in the African setting [15]. Results from the regression analysis suggest that other factors other than travel distance may contribute to late presentation and management. Anecdotally, an identified key barrier to care based on caregiver-surgeon interactions is financial constraints. As a result, parents may delay seeking care until their child’s condition becomes an emergency, necessitating urgent intervention. Other key obstacles further complicating access to timely care include sociocultural beliefs and misconceptions about the condition [15]. These factors also led to some caregivers delaying decision-making or declining surgical intervention for their children despite the neurosurgery team spending substantial time counseling parents/caregivers on hydrocephalus and CSF diversion. These patient-related factors together with systemic factors contributed to about 24% of patients not receiving surgical intervention.

The systemic complexities impacting access to hydrocephalus care include limited pediatric neurosurgery workforce availability, poor neurosurgical infrastructure, and a poor referral system. These factors contribute to not only delayed presentation at KATH but also delayed treatment. Less than 25% lived in the Kumasi metropolis where KATH is located. This implies that the majority of the patients might have first sought medical attention from peripheral facilities before they were referred to KATH for neurosurgical management. Delays in diagnosis and referral at these peripheral facilities often in rural areas where there is little to no neurosurgical expertise may also contribute to late presentation at KATH [16].

The median time from initial presentation to surgical treatment in the overall cohort was 29 days, which is relatively longer than the timelines reported in other African treatment centers (9.5–23 days) [13, 17, 18].

At KATH, VPS and ETV ± CPC are the primary hydrocephalus treatment options. All endoscopic procedures were, however, carried out by only one neurosurgeon (FNB) who has endoscopic expertise, while the other two neurosurgeons (AL and BO) and neurosurgery trainees only offered VPS. This limitation likely influenced the relatively higher proportion of patients with VPS observed in the cohort, as well as the relatively longer surgery wait times for patients in the ETV ± CPC cohort due to the backlog of cases. From our results, seven patients died while awaiting surgical intervention, which may have been contributed to by the long surgery wait times. With only 1 day set aside each week for all pediatric neurosurgical cases, cases other than hydrocephalus including emergent cases compete for operating room time which further impact wait times even after a surgical decision is made. The observed slight reduction in time to CSF diversion for patients with a greater travel distance in the regression analysis suggests that patients from farther areas were prioritized in surgery scheduling to help alleviate their travel burden.

In the 30-day postoperative period, seizures and shunt infections were the most frequently reported complications. The 8% surgical mortality reported is comparable to reports from other SSA countries (5.5%–8%) [14, 17, 19]. Morbidity and mortality are likely multifactorial, driven by surgical, systemic, and patient factors. These include surgical technique, prolonged operative times, poor nutritional status of patients, and disease severity [17, 19, 20]. Patient and maternal parental factors include the underlying cause of hydrocephalus, malnutrition, severity of disease, young maternal age, and low literacy rates [19, 21]. These same factors likely contributed to the extended postoperative length of stay.

Our results suggest relatively low re-intervention rates which we believe are partly related to challenges with patient follow-up and obtaining surveillance imaging in the follow-up setting. The time to failure in both VPS and ETV ± CPC cohorts was similar and occurred in less than 6 months. This contrasts with HIC studies which report that ETV failures occur within the first 6 months whereas there is a 2-year median time to shunt failure [22–25]. There was however no survival difference between the VPS and ETV ± CPC cohorts similar to other studies [14, 26]. Caregivers’ inability to recognize warning signs and symptoms of treatment failure contributes to delays in seeking follow-up care and may result in avoidable deaths [19]. This may partially explain the relatively low re-intervention rates and postoperative complications reported.

Although follow-up was achieved in 92% of patients, medium- to long-term surveillance remains a major challenge due to multiple factors including the cost of neuroimaging which is covered by patients out of pocket [8]. Multiple strategies including telephone follow-up and home visits conducted by the CURE Neuro Care Program Coordinator where feasible have been used by the neurosurgical team to improve follow-up rates. These approaches are constrained by resource limitations. Limitations to these alternate approaches to follow-up include the long travel distance and poor telecommunication network coverage in remote towns. Also, there may be limited or inaccurate information from caregivers to guide clinical decision-making. The high mortality rate is also a reflection of sociocultural and religious beliefs and societal stigma leading to child neglect and abandonment depriving the patients of neurosurgical care in some cases [27, 28].

### Limitations

This study has several limitations, many of which are inherent to descriptive studies. Data collection was particularly challenging due to resource constraints. The CURE Neuro Care Program Coordinator, who played a central role in data acquisition, had multiple responsibilities that limited the time available for comprehensive data collection. Additionally, the poor clinic follow-up contributed to significant data gaps. To mitigate this, an intern assisted with some follow-up data collection by contacting caregivers via phone which is not without recall bias or potential inaccuracies especially given the low literacy levels among caregivers. Another key limitation was the inability to ascertain the exact cause of mortalities. Not all deaths may have been hydrocephalus-related and could have been related to other factors including comorbid conditions. This study also did not explore factors associated with delayed presentation and care and their complex interactions influencing these delays.

### Future directions and recommendations

KATH has made progress in improving access to hydrocephalus and spina bifida care through both institutional and international support from the Spina Bifida and Hydrocephalus Foundation of Ghana and Child Help International. These include the provision of a free “House of Hope” hostel facility for all patients and caregivers and Chhabra Surgiwear system for patients undergoing VPS [10]. While the above efforts serve as a good foundation for improving access to hydrocephalus care, there is a tremendous need for additional capacity building support and continuous quality improvement efforts aimed at decreasing delays in presentation and surgical treatment to improve hydrocephalus outcomes. These include a dedicated operating room space for pediatric cases to help decrease surgical wait times. Beyond paying for neuroimaging out of pocket, the cost of surgery and hospitalization is cost-shared by patients under the National Health Insurance Scheme (NHIS) [8]. The NHIS of Ghana does not eliminate significant out-of-pocket cost for pediatric neurosurgical conditions. There is a need for an expansion of the insurance scheme to help reduce the financial burden on caregivers and vulnerable populations [29]. Implementation of public health education programs is essential to raise awareness, address misconceptions, and promote timely health-seeking behaviors. A national policy mandating a food fortification policy and folate supplementation for women of childbearing age could significantly reduce the incidence of myelomeningocele, which is currently the leading cause of hydrocephalus in this setting. Policy interventions should also prioritize other primary prevention measures including neonatal infection control and standardized treatment protocols to reduce CNS-related complications. Improving the referral system and communication between KATH and peripheral healthcare facilities is also necessary as these facilities often serve as the first point of contact for patients but may lack the training and resources to identify and refer hydrocephalus cases promptly. Future efforts should aim at improving follow-up care and data collection systems to ensure a more robust dataset for future studies and a better understanding of factors impacting treatment outcomes.

## Conclusion

Pediatric hydrocephalus presents a significant disease burden in KATH, Kumasi-Ghana, with majority of patients traveling long distances for their hydrocephalus care. Despite limited follow-up, ETV ± CPC and VPS demonstrated similar treatment success. Public health interventions targeting prevention and provision of timely neurosurgical care may help reduce disease burden and improve outcomes.

## Data Availability

No datasets were generated or analysed during the current study.
